# Healthier teams, safer care: workforce-driven determinants of quality of care and patient safety

**DOI:** 10.1093/eurpub/ckaf124

**Published:** 2026-01-23

**Authors:** David Berhanu, Miglė Trumpickaitė, Lenio Capsaskis, Válter R Fonseca, Yanina Andersen, Álvaro Cerame, Tomas Zapata, João Breda

**Affiliations:** European Junior Doctors Association, Brussels, Belgium; Faculdade de Medicina, Universidade de Lisboa, Lisbon, Portugal; Lisbon Clinical Academic Center (CCAL), Lisbon, Portugal; European Junior Doctors Association, Brussels, Belgium; WHO Office on Quality of Care and Patient Safety, WHO Regional Office for Europe, Athens, Greece; WHO Office on Quality of Care and Patient Safety, WHO Regional Office for Europe, Athens, Greece; WHO Regional Office for Europe, Copenhagen, Denmark; European Junior Doctors Association, Brussels, Belgium; WHO Regional Office for Europe, Copenhagen, Denmark; WHO Office on Quality of Care and Patient Safety, WHO Regional Office for Europe, Athens, Greece

Globally, inadequate quality of healthcare provision is a major driver of excess mortality [1]. Of the 8.6 million deaths from treatable conditions, in low- and middle-income countries, around 60% are due to poor-quality care [1]. Across WHO European Region, significant gaps in quality of care and patient safety persist [2]. Addressing between-hospital variation has been associated with prevention of hospital deaths, readmissions, and long patient stays [3]. Improving patient safety has the potential to save more than 3 million lives annually [4]. Up to 10% of patients are subject to an adverse event in healthcare [5]. At the same time, health systems are struggling with workforce shortages and increased demand from an ageing population with a longer lifespan [2, 6, 7]. These shortages have been linked to deteriorating working conditions and psychosocial well-being of medical professionals, increasingly recognized as factors impacting quality of care and patient safety [8, 9]. Research shows that decreased well-being and poor working conditions of health workers are associated with increased burn-out, medical errors, worse patient-provider engagement, and poorer health outcomes [9]. As such, health workforce must be addressed to improve quality of care and patient safety and achieve Universal Health Coverage. The recent WHO/Europe report, ‘Taking the pulse of quality of care and patient safety in the WHO European Region: multidimensional analysis and future prospects’, mapping the quality of care in the 53 member states of the European region underlines the importance of workforce for high-quality care. Indicators addressing the interplay between medical professionals and quality outcomes can further strengthen quality of care and patient safety at the health system level [2].

In November 2024, the European Junior Doctors’ Association (EJD) and World Health Organization Regional Office for Europe co-organized a 2-day workshop titled ‘Healthier Teams, Safer Care’. This workshop brought together experts to discuss priorities for junior medical doctors across the region by facilitating dialogue, enhancing collaboration and joint planning. It aimed to bridge workforce-related issues and their impact on quality of care and patient safety.

During the workshop, there was a consensus that workforce is decisive for quality outcomes (Table 1). Quality of care and patient safety post-graduate training emerged as a strong element to ensure ongoing competency and alignment with updated evidence-based good practices. Discussions also emphasized the critical role of a quality culture within the broader context of health systems to involve medical professionals in a learning and non-punitive environment.

**Table 1.** Key takeaways from the “Healthier Teams, Safer Care” workshop

**Key Messages**	**Centrality of workforce on quality outcomes**
	The situation of the workforce significantly impacts quality of care (QoC), linking workforce burnout and dissatisfaction to increased medical errors, worse patient-physician communication, and poorer healthcare system outcomes. The well-being of medical workers is a cornerstone for safer care and better healthcare outcomes, demanding urgent attention from policymakers and organizations.
	**Patient safety and QoC training**
	A non-punitive and supportive culture is essential to encourage error reporting and implement practices that reduce risks, enhancing patient safety. Patient safety and QoC principles must be foundational in PGT curricula and reinforced throughout a physician’s and other health professionals’ career (CME/CPD). Fostering multidisciplinary teamwork and collaboration through structured interdisciplinary training, which strengthens healthcare delivery, improves patient outcomes, and increases efficiency across the system.
	**Developing standardized indicators for monitoring**
	Harmonized metrics for workforce-related conditions are needed to monitor the well-being of medical professionals and its impact on quality. These should include including indicators for: Working conditions—monitoring resting times and working hours can provide a useful insight to the working conditions of the health workforce and their potential impact on patient safety. Burnout—rates of burnout correlate with QoC outcomes and could provide valuable insights into the wellbeing of the workforce and its direct impact on care quality Retention rates—high staff turnover has a detrimental impact on QoC and continuity of quality practices and safety protocols. Therefore, analysing the ability of a system to retain its professionals can be used as a proxy of the quality of the teams, institutions and care provided. Standardized training and reporting systems—assessing the implementation of mandatory patient safety training for medical professionals can provide an insight into the standard practices and quality culture. Additionally, monitoring the existence and usage rate of reporting systems that empower workers to suggest safety improvements and to prevent errors may be a useful indicator.
	**Policy and stakeholder engagement**
	European health stakeholders must prioritize workforce well-being and develop policies that invest in better working conditions as a key measure to improving QoC.

CME, continuous medical education; CPD, continuing professional development; PGT, post-graduate training; QoC, quality of care.

The dialogue stressed the importance of creating a set of harmonized indicators for measuring workforce-related parameters from a quality of care and patient safety perspective. Working hours and resting times have a well-established correlation with medical errors and a detrimental impact on quality of care [10, 11]. Similarly, the burnout rates negatively impact patient care and could provide insight into the psychological health and wellbeing of the workforce [12]. The existence of standardized quality of care and patient safety training and reporting systems could also be used as a quality improvement and safety culture proxy. The lack of patient safety training for medical professionals contributes to underreporting of errors, usually driven by a punitive culture and mistrust in reporting systems [13]. Finally, retention rates could be a useful surrogate for both the wellbeing and satisfaction of the workforce and the quality-of-care provision. Higher staff turnovers prevent continuity of care and may prevent integrated and safe practices, compromising quality of care and increasing costs [14].

The well-being of the medical workforce, including doctors at all stages of their careers, is central to achieving safer care and high-quality services. Health systems governance mechanisms are encouraged to incorporate workforce metrics to assess and guide quality of care and patient safety interventions (Table 1). Investing in better working conditions will improve motivation, retention, and mitigate the scarcity of medical professionals, with accompanying improvements in quality and population health outcomes. Policymakers need to act upon workforce scarcity, motivation and job satisfaction, continuous education, and workplace culture, to make strides in quality of care and patient safety.

## Data Availability

No new data were generated or analysed in support of this research.
